# A novel type bacterial flagellar motor that can use divalent cations as a coupling ion

**DOI:** 10.1038/srep19773

**Published:** 2016-01-22

**Authors:** Riku Imazawa, Yuka Takahashi, Wataru Aoki, Motohiko Sano, Masahiro Ito

**Affiliations:** 1 Graduate School of Life Sciences Toyo University, Oura-gun, Gunma 374-0193, Japan; 2 Bio-Nano Electronics Research Centre, Toyo University, 2100 Kujirai, Kawagoe Saitama 350-8585, Japan; 3 Faculty of Life Sciences, Toyo University, Oura-gun, Gunma 374-0193, Japan

## Abstract

The bacterial flagellar motor is a sophisticated nanomachine embedded in the cell envelope and powered by an electrochemical gradient of H^+^, Na^+^, or K^+^across the cytoplasmic membrane. Here we describe a new member of the bacterial flagellar stator channel family (MotAB1 of *Paenibacillus* sp. TCA20 (TCA-MotAB1)) that is coupled to divalent cations (Ca^2+^and Mg^2+^). In the absence of divalent cations of alkaline earth metals, no swimming was observed in *Paenibacillus* sp. TCA20, which grows optimally in Ca^2+^-rich environments. This pattern was confirmed by swimming assays of a stator-free *Bacillus subtilis* mutant expressing TCA-MotAB1. Both a stator-free and major Mg^2+^uptake system-deleted *B. subtilis* mutant expressing TCA-MotAB1 complemented both growth and motility deficiency under low Mg^2+^conditions and exhibited [Mg^2+^]_in_ identical to that of the wild-type. This is the first report of a flagellar motor that can use Ca^2+^and Mg^2+^as coupling ions. These findings will promote the understanding of the operating principles of flagellar motors and molecular mechanisms of ion selectivity.

The bacterial flagellar motor is embedded in the cell envelope, and is usually powered by an electrochemical gradient of protons (H^**+**^), sodium (Na^**+**^), or potassium (K^**+**^) across the cytoplasmic membrane^1^,^2^. MotAB-type stators use H^**+**^as the coupling ion, whereas MotPS- and PomAB-type stators use Na^**+**^. *Bacillus subtilis* employs H^**+**^-coupled MotAB and Na^**+**^-coupled MotPS stators to generate the torque required for flagellar rotation^2^,^3^,^4^.

The Mot complexes contain channels that use either H^**+**^or Na^**+**^, with some bacteria having only one type and others having two distinct types with different ion-coupling^5^,^6^. However, in 2008, alkaliphilic *Bacillus clausii* KSM-K16 was identified as the first bacterium with a single stator-rotor that uses both H^**+**^and Na^**+**^ for ion-coupling at different pH ranges. Mutations that convert the bifunctional stator to each single stator type have been demonstrated, and the same approach was applied to confer dual-ion use on the two single ion-use stators of *B. subtilis*^7^. Subsequent findings have shown that alkaliphilic *Bacillus alcalophilus* AV1934 uses Na^**+**^, K^**+**^, and Rb^**+**^as coupling ions for flagellar rotation^1^.

We considered that calcium ions, existing abundantly in nature, are one of the next candidates of coupling ions of the bacterial flagellar motor. Although the role of Ca^2**+**^in eukaryotes has been widely characterized, its role in prokaryotes is not completely understood. Ca^2**+**^in prokaryotes is involved in the maintenance of cell structure, chemotaxis, transport, and cell differentiation processes, including sporulation, heterocyst formation, and fruiting body development^8^,^9^,^10^. However, divalent cation-coupled flagellar motors have not yet been identified in nature. Therefore, we isolated a bacterium (*Paenibacillus* sp. strain TCA20) that showed Ca^2 + ^-dependent growth from a water sample collected from Tsurumaki-Onsen (latitude and longitude: 35.387668 N 139.277898 E), a well-known Japanese hot spring in Kanagawa Prefecture, Japan, which contains a high Ca^2+^ concentration (1,740 mg/l; approximately 44 mM). Recently, we reported the draft genome sequence of this bacterium^11^. Here we characterize its bacterial flagellar motor and report a novel bacterial flagellar stator that can use both Mg^2+^ and Ca^2+^ as coupling cations for flagellar rotation.

## Results and Discussion

### Isolation and characterization of *Paenibacillus* sp. TCA20 motility

**Figure 1:**
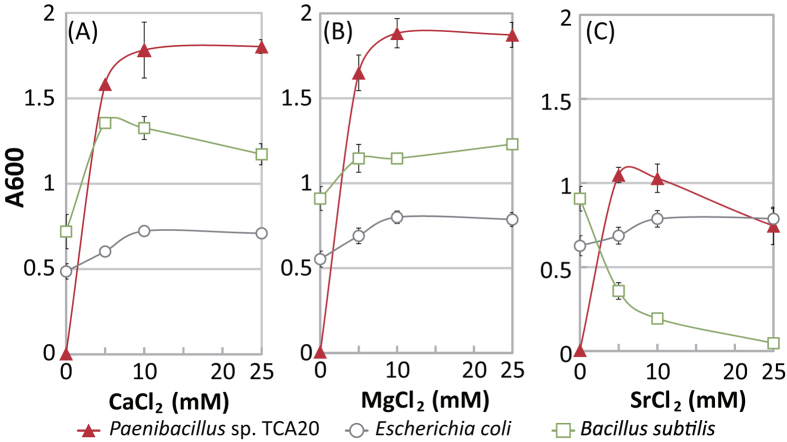
Effect of divalent cations on growth of *Paenibacillus*sp. TCA20, *E. coli*, and *B. subtilis.* Growth in Tris medium containing several CaCl_2_ (**A**), MgCl_2_ (**B**), or SrCl_2_ (**C**) concentrations was monitored at A_600_. The results are the averages of three independent experiments, with error bars representing standard deviations.

**Figure 2:**
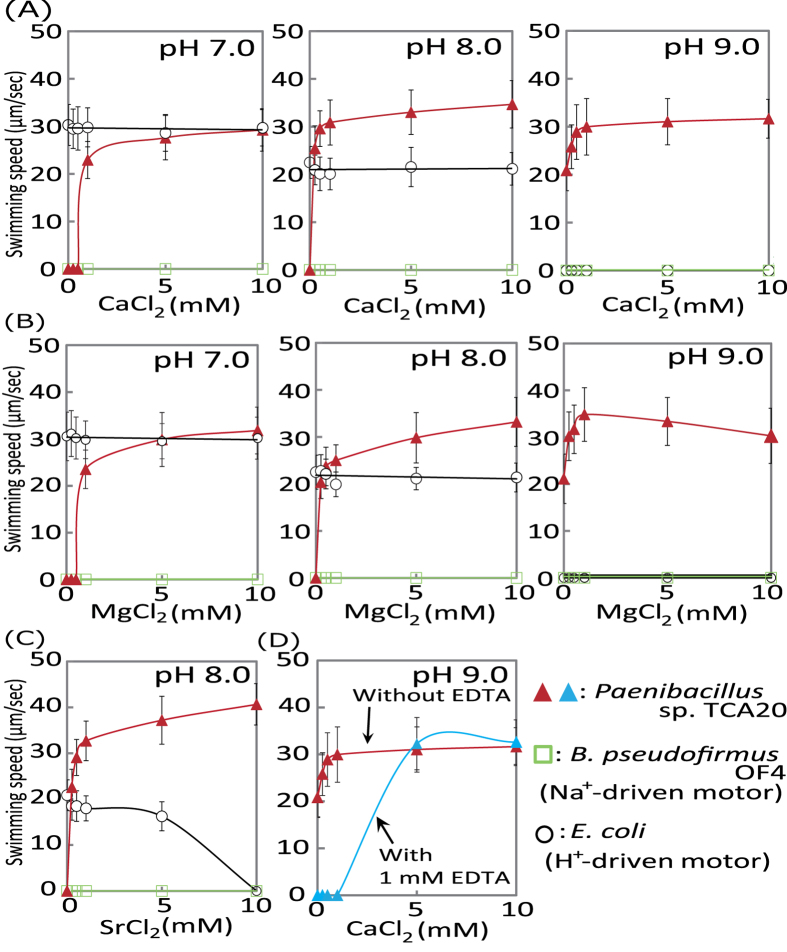
Effect of divalent cations on swimming speed of *Paenibacillus* sp. TCA20, *E. coli*, and *B. pseudofirmus*OF4. Swimming speeds of *Paenibacillus* sp. TCA20, *E. coli*, and *B*. *pseudofirmus* OF4 cells were measured in 30 mM Tris-HCl containing 5 mM glucose and several CaCl_2_ (**A**), MgCl_2_ (**B**), or SrCl_2_ (**C**) concentrations. The results represent the average swimming speed of 30 independent cells from three independent experiments. The error bars indicate standard deviations. (**D**) The swimming speed of *Paenibacillus* sp. TCA20 cells was measured of in 30 mM Tris-HCl with/without 1 mM EDTA at pH 9.0.

**Figure 3:**
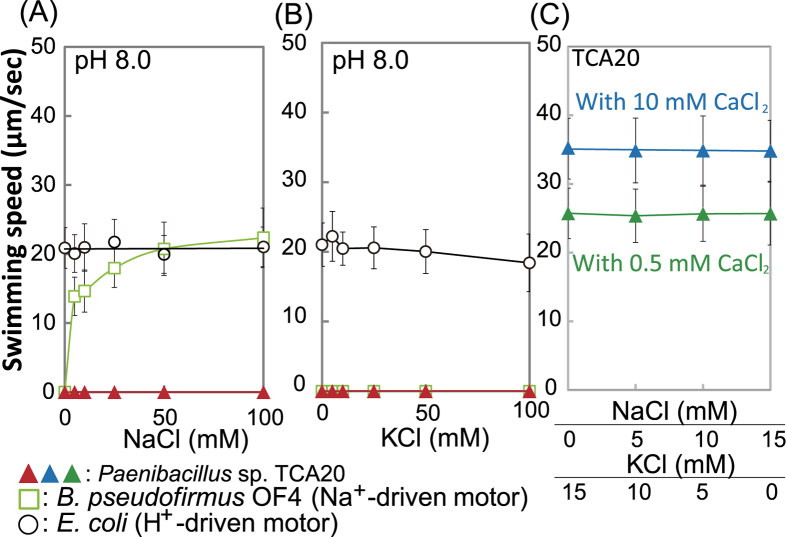
Effect of monovalent cations on swimming speed of *Paenibacillus*sp. TCA20, *E. coli*, and *B. pseudofirmus* OF4. Swimming speeds of *Paenibacillus* sp. TCA20, *E. coli*, and *B*. *pseudofirmus* OF4 cells were measured in 30 mM Tris-HCl containing 5 mM glucose and several NaCl (**A**) or KCl (**B**) concentrations. The relationship between swimming speed in 30 mM Tris-HCl containing the various indicated CaCl_2_, KCl, and NaCl concentrations is shown in (**C**). The results represent the average swimming speeds of 30 independent cells from three independent experiments. The error bars indicate standard deviations.

*Paenibacillus* sp. TCA20 requires >5 mM divalent cations of an alkaline earth metal, including Ca^2+^, magnesium (Mg^2+^), or strontium (Sr^2+^), for growth (Fig. 1). On the other hand, *E. coli* and *B. subtilis* showed no requirement of such an alkaline earth metal for growth. Adding Ca^2+^ and Mg^2+^ to the medium moderately enhanced their growth was better than without divalent cations, even though the growth of *B. subtilis* was inhibited by Sr^2+^. Swimming behavior of this bacterium showed Ca^2+^- and Mg^2+^-dependent motility at pH less than 8.0 and Sr^2+^-dependent motility at pH 8.0 (Fig. 2A–C and S1). However, swimming (approximately 20 μm/s) of strain TCA20 was observed in the absence of divalent cations at pH 9.0. Furthermore, no motility was observed when 1 mM ethylenediaminetetraacetic acid (EDTA) was added to the motility assay buffer (pH 9.0) and >1 mM CaCl_2_ in the same buffer was required for Ca^2+^-dependent motility (Fig. 2D). No swimming was observed when up to 100 mM Na^+^ and/or K^+^ was added to the buffer at pH 8.0 (Fig. 3A,B). Additional Na^+^ and K^+^ in the swimming assay buffer did not stimulate Ca^2+^-dependent motility (Fig. 3C). In the presence of elevated divalent cations, no stimulation was observed in the swimming velocity of *Escherichia coli*, which has a H^+^-coupled motor at pH 7 and 8, and no swimming was observed at pH 9.0 (Fig. 2 and S1). Alkaliphilic *Bacillus pseudofirmus* OF4, which has a Na^+^-coupled motor, did not exhibit swimming behavior under these conditions.

At low concentrations of divalent cations, the Ca^2+^- and Mg^2+^-dependent swimming velocity by the flagellar motor was increased by elevating the pH (Fig. 2A,B and S1A,B). At pH 9.0, despite the absence of divalent cations, swimming was observed. However, when the chelating reagent EDTA was added, no swimming was observed up to 1 mM CaCl_2_ (Fig. 2D). Therefore, we investigated the possibility of carry-over of Ca^2+^ in the buffer and found that it contained 0.69 mM, which may explain why motility was observed with presumably no divalent cations at pH 9.0. These results suggest the possibility of using Ca^2+^, Mg^2+^, and Sr^2+^ as coupling cations for flagellar rotation of strain TCA20.

### Identification of TCA-MotAB1 and TCA-MotAB2

The draft genome of strain TCA20 was sequenced and deposited in the DDBJ/EMBL/GenBank databases (accession number BBIW00000000.1). The annotation of the draft genome sequence shows that strain TCA20 has two sets of MotA/MotB-like genes: TCA-motA1/motB1 and TCA-motA2/motB2, respectively. TCA-MotA1 (GenBank: GAK41226.1, 264 aa) and MotB1 (GenBank: GAK41227.1, 254 aa) showed moderate resemblance to *B. subtilis* MotA (270 aa) and MotB (261 aa) that constitute the H^+^-coupled *B. subtilis* stator Mot complex (37% and 31% identity, and 61% and 52% similarity, respectively) and to *B. subtilis* MotP (272 aa) and MotS (242 aa) that constitute a Na^+^-coupled *B. subtilis* stator Mot complex (36% and 32% identity and 60% and 51% similarity, respectively). TCA-MotA2 (GenBank: GAK43333.1, 267 aa) and MotB2 (GenBank: GAK43334.1, 264 aa) were closely related to *B. subtilis* MotA and MotB (53% and 44% identity, and 72% and 64% similarity, respectively).

In *B. subtilis*, the Ca^2+^-specific Ca^2+^ /H^+^ antiporter ChaA and the P-type Ca^2+^-transporting ATPase YloB were identified^12^,^13^, suggesting that ChaA and YloB are important for Ca^2+^ signaling during sporulation or germination. The annotation of the draft genome sequence showed that *Paenibacillus* sp. strain TCA20 has a *chaA* gene (AN: GAK39775) and a gene that encodes a putative P-type Ca^2+^-transporting ATPase (AN: GAK39789)^11^. In *Streptococcus pneumonia*, the P-type Ca^2+^-transporting ATPase CaxP is used to avoid Ca^2+^ accumulation in the eukaryotic host^14^. In addition to Ca^2+^ signaling during sporulation or germination these transporters may be important for the growth of strain TCA20 under high Ca^2+^ concentrations.

**Figure 4:**
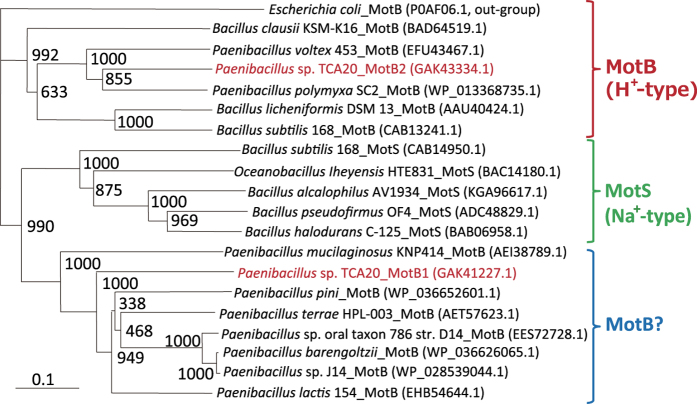
Phylogenetic tree of the stator subunits MotB and MotS of the flagellar motor from *Paenibacillus*, *Oceanobacillus*, and *Bacillus* sp. using the neighbor-joining method. The MotB sequence of *E. coli* was used as an outgroup. Bar, 0.1 substitutions per amino acid position. The phylogenetic tree was constructed with ClustalW (http://clustalw.ddbj.nig.ac.jp) with 1,000 bootstrap samplings. *Paenibacillus* sp. TCA20 is shown in red text.

A phylogenetic tree of the stator proteins of the flagellar stator subunits MotB and MotS from *Paenibacillus*, *Oceanobacillus*, and *Bacillus* sp. and *E. coli* is shown (Fig. 4). The phylogenetic features of two stator proteins suggest that TCA-MotAB2 functions as an H^+^-type stator. However, TCA-MotAB1 belongs to a different stator cluster from the H^+^-coupled MotAB or Na^+^-coupled MotPS complex (Fig. 4). Functional analysis of the cluster motility containing TCA-MotB1 has not yet been experimentally characterized, although each protein was automatically annotated as MotB in the database. Strains belonging to this cluster were isolated from various environments (e.g., soil, oral swab from patient, rhizosphere of plant, and assembly facility), and had no significant commonality^15^,^16^,^17^,^18^ *Paenibacillus lactis* 154 belonging to this cluster was isolated from milk, which is rich in Ca^2+^ 19. Although *P. lactis* 154 was isolated from a Ca^2+^-abundant environment, the identity and similarity between TCA-MotB1 and MotB from *P. lactis* 154 were only 61% and 76%, respectively. A protein BLAST search returned no protein with homologies of >68% and >63% against TCA-MotA1 and TCA-MotB1, suggesting unique features of the TCA-MotAB1 stator complex.

**Figure 5:**
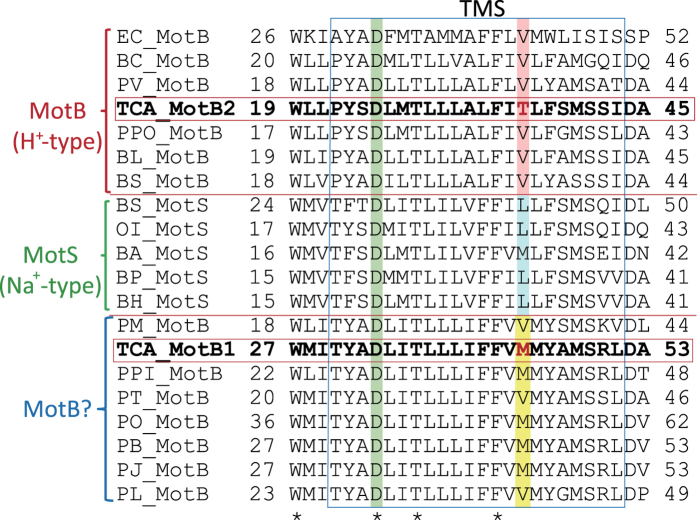
Multiple alignment of the regions containing the single transmembrane segment of stator proteins of the flagellar motor from *Paenibacillus*, *Oceanobacillus*, and *Bacillus* sp. Alignments of the regions containing the single transmembrane segment of *Paenibacillus* sp. TCA20 (TCA_MotB1, TCA-MotB2), *E. coli* (EC_MotB), *B. clausii* (BC_MotB), *P. vortex* (PV_MotB), *P. polymyxa* (PPo_MotB), *B. licheniformis* (BL_MotB), *B. subtilis* (BS_MotB), *B. subtilis* (BS_MotS), *Oceanobacillus iheyensis* (OI_MotS), *B. alcalophilus* (BA_MotS), *B. pseudofirmus* OF4 (BP_MotS), *B. halodurans* C-125 (BH_MotS), *P. mucilaginosus* (PM_MotB), *P. pini* (PPi_MotB), *P. terrae* (PT_MotB), *Paenibacillus* sp. Oral taxon 786 str. D14 (PO_MotB), *P. barengoltzii* (PB_MotB), *Paenibacillus* sp. J14 (PJ_MotB), and *P. lactis* (PL_MotB). The position of L41 in BS MotS (the eighth line from the top) is conserved among all MotS-Na^ + ^-type proteins, except BA_MotS, and is highlighted in light blue. The position of V43 in EC_MotB (the first line) is conserved among all MotB-H^ + ^-type proteins, except TCA_MotB2, and is highlighted in light pink. The position of M41 in TCA_MotB1 (the seventh line from the bottom) and its same cluster is highlighted in yellow. The same position in TCA_ MotB1 or TCA_MotB2 encodes methionine or threonine instead of the conserved valine residue shown in red. A universally conserved aspartic acid residue of MotB that is a predicted as an H^+^-binding site in *E. coli* is highlighted in green.

A critical amino acid residue for the coupling of ion selectivity exists in between a single transmembrane region of MotB and MotS^1^,^7^. The bacterial H^+^-coupled MotB-type stator subunits contain a conserved valine residue in between the transmembrane segment, except for TCA-MotB2 (alignment; Fig. 5). However, the Na^+^-coupled MotS-type stator subunits contain a conserved leucine residue at the same location, except for *B. alcalophilus* MotS (BA-MotS). Because of the M33L substitution, BA-MotS motility lost K^+^-dependence that became only Na^+^-dependence^7^. The same position in TCA-MotB1 and TCA-MotB2 contains a methionine and threonine residue, respectively. Because strain TCA20 was isolated from a unique environment, critical amino acid residues for ion selectivity in both stators may not be conserved. This apparent exception may result in future identification of critical amino acid residues for coupling ion selectivity.

### Stator-free *B. subtilis* mutants expressing TCA-MotAB1 and TCA-MotAB2

Because strain TCA20 was not genetically accessible, we used each single stator gene to directly compare the swimming properties conferred by TCA-MotAB1 and TCA-MotAB2, which were introduced into the *lacA* locus of *B. subtilis* strain ΔABΔPS from which both native BS-*motAB* (*motAB* of *B. subtilis*) and BS-*motPS* (*motPS* of *B. subtilis*) were deleted.

**Figure 6:**
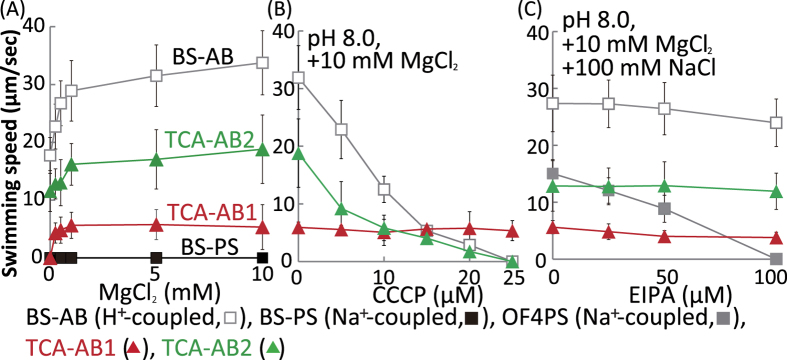
Effect of Mg^2+^, protonophore CCCP and Na^ + ^channel inhibitor EIPA on swimming speed of *B. subtilis* mutant strains. BS-AB, BS-PS, OF4PS, TCA-AB1, and TCA-AB2 mutant strains were grown for 6 h at 37 °C in Spizizen I medium plus 1 mM MgCl_2_ and 1% xylose at pH 8.0 with shaking. Cells were suspended in 1 ml of phosphate buffer (pH 8.0) (**A**), plus the indicated amounts of CCCP plus 10 mM MgCl_2_ (**B**), plus the indicated amounts of EIPA plus 10 mM MgCl_2 _and 100 mM NaCl (**C**), and then incubated at 37 °C for 10 min. Phosphate buffer contained 10 mM potassium phosphate (pH 8.0), 5 mM glucose, 1% xylose, 10 μg/ml tryptophan and lysine. The swimming speed is the average speed of >30 cells.

The resulting *B. subtilis* mutant strains expressing TCA-MotAB1 or TCA-MotAB2 were named TCA-AB1 and TCA-AB2, respectively. Each gene pair was controlled by a xylose inducible promoter (P_*xlyA*_). Both stators restored motility to the non-motile ΔABΔPS strain on soft agar plates containing 1% xylose. Furthermore, we determined the cations that are preferred for flagellar rotation by these mutants (Fig. 6A). The swimming speeds of TCA-AB1 and TCA-AB2 were measured at several Mg^2+^ concentrations in 10 mM potassium phosphate buffer (pH 8.0) with 5 mM glucose, 1% xylose, 10 μg/ml tryptophan and lysine. TCA-AB1 exhibited no swimming ability without added Mg^2+^ and exhibited stimulation by Mg^2+^. We performed the same experiment using CaCl_2_. However, the tumbling frequency of all strains was drastically increased and it was very difficult to measure the linear swimming velocity of each. Therefore, we tested the addition of only Mg^2+^ and found that the TCA-AB1 rotor prefers using Mg^2+^ in the heterologous neutralophilic host. BS-AB swimming, which has a distinct H^+^-coupled motor from *B. subtilis*, and TCA-AB2 were observed in the absence of Mg^2+^, and the speed was stimulated by increasing Mg^2+^ concentrations up to 10 mM (Fig. 6A). The stimulated phenotype of the swimming speed of a proton coupled motor by adding MgCl_2_ is of interest. The details of this mechanism are unknown. However, we speculated that proton entry by flagellar rotation is influence by intracellular pH homeostasis. The requirement of some amount of Mg^2+^ may facilitate proton circulation from inside and outside the cells and allow the influx of more protons by flagellar rotation. It is known that intracellular levels of metal ions of bacterium are carefully maintained by sensing of regulatory proteins and an RNA element^20^,^21^,^22^. No swimming was observed for strain BS-PS, which has a distinct Na^+^-coupled motor from *B. subtilis*, under these conditions.

Further, we tested the effects of several inhibitors on the bacterial flagellar motor. The protonophore carbonyl cyanide m-chlorophenyl hydrazone (CCCP), which dissipates electrochemical H^+^ gradients, did not affect TCA-AB1 swimming containing Mg^2+^ at inhibitor concentrations up to 25 μM at pH 8.0 (Fig. 6B). Conversely, TCA-AB2 swimming and H^+^-coupled BS-AB were completely inhibited by CCCP addition at 25 μM with/without Mg^2+^ at pH 8.0 (Fig. 6B). The Na^+^-coupled flagellar stator inhibitor 5-(N-ethyl-N-isopropyl)-amiloride (EIPA) did not affect the swimming of TCA-AB1 and TCA-AB2 containing 10 mM MgCl_2_ and 100 mM NaCl at inhibitor concentrations up to 100 μM (Fig. 6C). These results support that the coupling ions of the TCA-MotAB1 and TCA-MotAB2 stator complex are Mg^2+^ and H^+^, respectively. TCA20 motility was completely dependent on divalent cation concentrations. Therefore, the stator protein TCA-MotAB2 may not be expressed under these culture conditions.

### Growth and measurement of intracellular Mg^2+^ concentrations of a derivative strain of TCA-AB1 which was deleted in the major Mg^2+^ uptake system

Strain TCA-AB1 showed Mg^2+^-dependent motility. However, there was no direct evidence of actual inward translocation of Mg^2+^ through the stator protein TCA-MotAB1.

**Figure 7:**
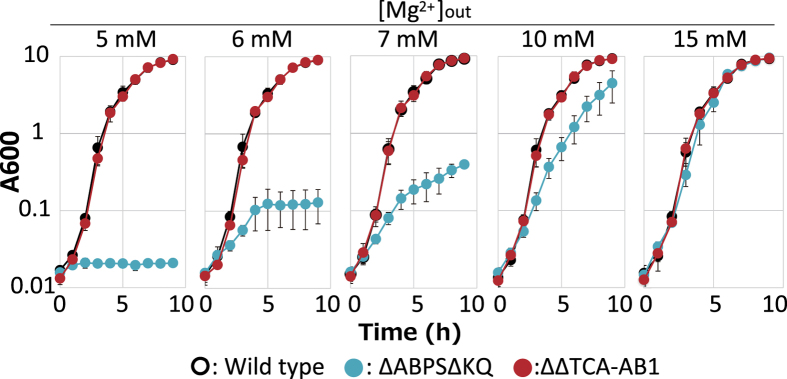
Effect of extracellular Mg^2 + ^concentrations on growth of *B. subtilis* and its Mg^2 + ^uptake system-deleted mutant strains. WT (*B. subtilis* BR151MA), ΔABPSΔKQ, and ΔΔTCA-AB1 strains were grown for 9 h at 37 °C in 2 × TY medium with 1% xylose plus MgCl_2_ (0, 1, 2.5, 5, or 10 mM) with shaking. Growth was assessed hourly at A_600_.

There are at least five distinct Mg^2+^ transporters [YkoK (also termed MgtE), YloB, YfjQ, YqxL, and CitM] in *B. subtilis*. Of these, YkoK and YfjQ are major Mg^2+^ uptake systems, and if both genes are deleted, the deleted strain requires >6 mM Mg^2+^ for growth (Fig. 7). We constructed strain ΔΔTCA-AB1, a derivative strain of TCA-AB1, and additionally deleted both *ykoK* and *yfjQ*. This strain required Mg^2+^ for growth without xylose. We hypothesized that if TCA-MotAB1 is incorporated into a functional flagellar motor and takes up Mg^2+^ during flagellar rotation, strain ΔΔTCA-AB1 will complement the Mg^2+^ growth requirement with xylose. Strain ΔABPSΔKQ, a derivative strain of ΔABΔPS with deletions of *ykoK* and *yfjQ*, was used as a negative control.

**Figure 8:**
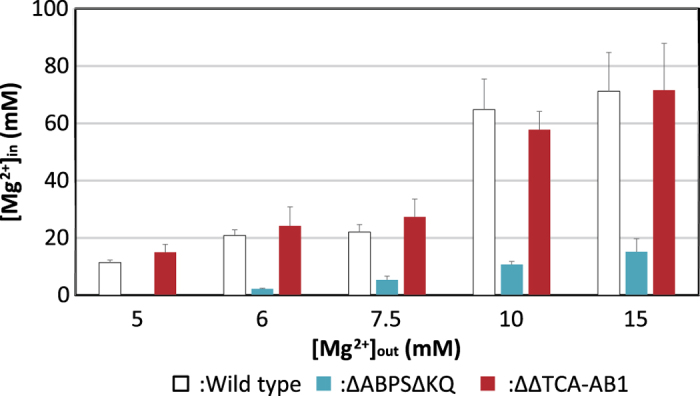
Effect of extracellular Mg^2 + ^concentrations on intracellular Mg^2 + ^concentration of *B. subtilis* and its Mg^2+^ uptake system-deleted mutant strains. WT (*B. subtilis* BR151MA), ΔABPSΔKQ, and ΔΔTCA-AB1 strains were grown for 9 h at 37 °C in 2 × TY medium with 1% xylose plus MgCl_2_ (0, 1, 2.5, 5, or 10 mM) with shaking. Mg^2+^ concentrations per cell were measured as described in the text. Error bars indicate standard deviations of means from three independent experiments per strain.

Growth curves and intracellular Mg^2+^ concentrations of strains BR151MA (wild-type, WT), ΔΔTCA-AB1, and ΔABPSΔKQ were measured at several extracellular Mg^2+^ conditions (Figs 7 and 8). Strain ΔABPSΔKQ required >6 mM Mg^2+^ for growth (Fig. 7), whereas the strain ΔABPSΔKQ intracellular Mg^2+^ concentration was lower than that of WT under all conditions. However, the growth curve and [Mg^2+^]_in_ of strain ΔΔTCA-AB1 was similar to those of WT, as expected. ΔΔTCA-AB1 motility was observed under all conditions with xylose. Akanuma *et al*. reported an intercellular total Mg^2+^ concentration of *B. subtilis* WT of approximately 70 mM 23, similar to our data (Fig. 8). These results suggest that flagellar rotation and Mg^2+^ uptake are coupled.

Stator protein TCA-MotAB1 has a universally conserved Asp-33 residue of MotB1 that is critical for motility and is a predicted H^+^-binding site in *E. coli*^24^ (Fig. 5). The crystal structure of the flagellar stator has not been determined, except for the C-terminal hydrophilic region of MotB and PomB^25^,^26^. Braun *et al*. predicted amino acid residue arrangement in the transmembrane segment of the MotA subunit by cross-linking experiments of the *E. coli* stator MotAB^27^. A coupling ion influx pathway is formed by the third and fourth transmembrane segments of the MotA subunit and a single transmembrane segment of the MotB subunit^27^. There is no additional negative charged amino acid residue near residue Asp-33 of TCA-MotB1 and the third and fourth transmembrane segments of TCA-MotA1, suggesting that divalent cations work as coupling ions for flagellar rotation of strain TCA20, although the predicted coupling ion-binding site was a single negatively charged side chain of an aspartic acid residue. Utilizing divalent cations for flagellar rotation, the membrane potential would be consumed two times faster than utilizing monovalent cations. Therefore, motor torque coupled with divalent cations compared with monovalent cations is of interest.

The discovery of a novel type flagellar motor that can use divalent cations as coupling ions shows the diversity of flagellar motor proteins. Recently, Takekawa *et al* reported that one of the earliest flagellar motor proteins in hyperthermophilic bacterium *Aquifex aeolicus* can be driven by Na^+^ for energy coupling^28^. The path of evolution of the conventional flagellar motor proteins from the earliest ones is still poorly understood. It will be interesting to elucidate the evolution of stator proteins in connection with their habitat environment.

A major finding of our study was that a single bacterial flagellar stator of *Paenibacillus* sp. TCA20 coupled motility to divalent cations, including Mg^2+^ and Ca^2+^. This finding may facilitate the identification of additional examples of divalent cation-coupling capacity among the increasing number of bacteria that exhibit motility in extreme environments. Altering the coupling ion selectivity of the stator in the laboratory is time-consuming. However, if the identification of stator genes of microorganisms growing in extreme environments is possible, application of the flagellar motor as a nanomachine can progress.

## Materials and Methods

### Bacterial strains, plasmids and growth conditions

The strains and plasmids used in this study are shown in Table S1. To measure Mg^2+^- and Ca^2+^-dependent growth capacities of *E. coli*, *Paenibacillus* sp. TCA20, and *B. subtilis*, we initially attempted to grow *E. coli* and *B. subtilis* in Luria broth (LB) medium. *Paenibacillus* sp. TCA20 cells were aerobically grown on 30 mM Tris medium (pH 7.7) containing 5 mM CaCl_2_ overnight at 37 °C. Tris medium contained 30 mM Tris base, 7 mM citric acid monohydrate, 0.05% (w/v) yeast extract, 50 mM glucose and 1% (v/v) trace elements^29^. *E. coli* and *B. subtilis* cells were grown in LB medium under the same conditions. The culture was then inoculated into 30 mM Tris medium (pH 7.7) containing several different MgCl_2_, CaCl_2_, or SrCl_2_ concentrations at an absorbance of 0.01 at 600 nm (A_600_), and grown aerobically at 37 °C for 14 h. Growth was monitored by measuring the absorbance at A_600_.

The effects of increasing MgCl_2_ concentrations (i.e., 0, 1, 2.5, 5, or 10 mM) on growth of *B. subtilis* 168 strains BR151MA (WT), ΔABPSΔKQ, and ΔΔTCA-AB1 were determined in 2 × TY medium with 1% xylose at 37 °C with shaking and measured at A_600_.

### Plasmid construction

Selected *mot* genes were integrated into the chromosomal *lacA* locus of mutant strains under the control of the xylose-inducible P_*xylA*_ promoter using the plasmid pAX01. For the construction of a plasmid carrying intact *TCA-motAB1* and *TCA*-*motAB2* genes under the control of the P_*xylA*_ promoter, sets of primers were designed with nucleotides encoding *Sac*II sites. All primer sequences are provided in Table S2. Each amplified fragment was cloned into *Sac*II-digested pAX01, yielding PxylA-AB1 and PxylA-AB2, respectively. Each plasmid was integrated into the *lacA* locus of a *B. subtilis* ΔABΔPS host. Recombinant transformants were selected using conventional techniques to confirm the correct sequence of the insert.

### Measurement of swimming speed

For the measurement of swimming speed, *Paenibacillus* sp. TCA20 cells were aerobically grown on Tris medium (pH 7.7) plus 5 mM CaCl_2_ overnight at 37 °C. The culture was then inoculated into 20 ml of fresh Tris medium (pH 7.7) at an A_600_ of 0.01 and aerobically grown at 37 °C. *E. coli* cells were grown in LB medium under the same conditions. *B. pseudofirmus* OF4 cells were grown in alkaline complex medium under the same conditions^7^. BS-AB, BS-PS, TCA-AB1, and TCA-AB2 mutant strains were grown in Spizizen I medium^30^ plus 1 mM MgCl_2_ and 1% xylose at pH 8.0 under the same conditions. Spizizen I medium contained Spizizen salts, 0.5% glucose, 0.02% casamino acids, 0.1% yeast extract, 10 μg/ml tryptophan and lysine. Spizizen salts contained 85 mM K_2_HPO_4_, 40 mM KH_2_PO_4_, 15 mM (NH_4_)_2_SO_4_, 6 mM sodium citrate and 0.8 mM MgSO_4_. Highly motile cells in the late logarithmic phase were harvested by filtration on OMNIPORE membrane filters (0.45 μm) and washed three times with 2 ml of 30 mM Tris-HCl buffer that contained 5 mM glucose, and the indicated amounts of NaCl, KCl, CaCl_2_, MgCl_2_, SrCl_2_ and/or EDTA were used at the indicated pH values. Cells were suspended in 1 ml of the same buffer and incubated at 30°C for 10 min. For the measurement of swimming speed, *B. subtilis* and its derivative mutants were grown for 6 h at 37 °C in Spizizen I medium^30^ plus 1 mM MgCl_2_ and 1% xylose at pH 8.0 with shaking. Cells were suspended in 1 ml of phosphate buffer (pH 8.0) and the indicated amounts of CCCP, EIPA, MgCl_2_ and NaCl, and then incubated at 37 °C for 10 min. Phosphate buffer contained 10 mM potassium phosphate (pH 8.0) plus 5 mM glucose, 1% xylose, 10 μg/ml tryptophan and lysine. Cell motility was observed under a dark-field microscope using a Leica DMRE microscope and recorded in high definition with a digital color camera (model DFC310FX; Leica, Tokyo, Japan). Swimming speed was determined with particle tracking velocimetry software (DigiMo, Tokyo, Japan). The ionic strength of the assay buffers increased with the cation concentration. All results are the averages of three independent experiments in which the speed of 30 different cells was measured.

### Construction of Δy*koK* and Δ*yfjQ* mutants

The Δ*ykoK* and Δ*yfjQ* mutants were constructed by gene splicing via overlap extension as described previously^31^. For the construction of a fragment upstream and downstream of *ykoK*, two independent PCR reactions were performed on WT DNA with the primer sets YkoK-cm-1 and YkoK-cm-2 (i) and YkoK-cm-3 and YkoK-cm-4 (ii). The PCR products were used as templates for a second PCR with primers YkoK-cm-1 and YkoK-cm-4. The purified products were cloned into *Sma*I-digested pUC18Tc, yielding pUC18Tc-ΔykoK.

For the construction of a fragment upstream and downstream of *yfjQ*, two independent PCR reactions were performed on WT DNA with the primer sets YfjQ-cm-1 and YfjQ-cm-2 (i) and YfjQ-cm-3 and YfjQ-cm-4 (ii). The PCR products were used as templates for a second PCR with primers YfjQ-cm-1 and YfjQ-cm-4. The purified products were cloned into *Sma*I-digested pUC18Tc, yielding pUC18Tc-ΔyfjQ.

Strain ΔABPSΔKQ was constructed using the gene replacement approach described elsewhere^32^ and the *ykoK* and *yfjQ* region deletion of the *B. subtilis* ΔABΔPS host was confirmed by PCR.

### Measurement of intracellular Mg^2+^ concentration

*B. subtilis* BR151MA, ΔΔTCA-AB1, and ΔABPSΔKQ cells were grown at 37 °C in 2 × TY medium with 1% xylose plus MgCl_2_ (0, 1, 2.5, 5, or 10 mM) with shaking. The cells in the late logarithmic growth phase were harvested by centrifugation (8,000 rpm, 10 min, 25°C), such that there were an equal number of bacterial cells, and washed by suspension in 10 ml of 300 mM sucrose solution. Then, the cells were resuspended in 10 ml of 300 mM sucrose solution with 100 mM hydrochloric acid (HCl) solution. After shaking for 10 min, cell debris was removed by centrifugation and Mg^2+^ was measured by MG Metallogenics Mg^2+^ measurement LS (Metallogenics Co., Ltd., Chiba, Japan) that was calibrated with standard Mg^2+^ solutions of known concentrations. Intracellular Mg^2+^ was calculated by assuming that a *B. subtilis* cell is a cylinder (radius, 0.4 μm; length, 4 μm)^23^.

## Additional Information

**How to cite this article**: Imazawa, R. *et al.* A novel type bacterial flagellar motor that can use divalent cations as a coupling ion. *Sci. Rep.* **6**, 19773; doi: 10.1038/srep19773 (2016).

## Supplementary Material

Supplementary Information
