# Supplementary material for: A novel type bacterial flagellar motor that can use divalent cations as a coupling ion
Source: Sci Rep. 2016 Jan 22;6:19773. doi: 10.1038/srep19773 (PMC4726428; doi:10.1038/srep19773)
Supplement: Supplementary Information [file srep19773-s1.pdf]

supplementary information

# A novel type bacterial flagellar motor that can use divalent cations as a coupling ion

Riku Imazawa, Yuka Takahashi, Wataru Aoki, Motohiko Sano and Masahiro Ito

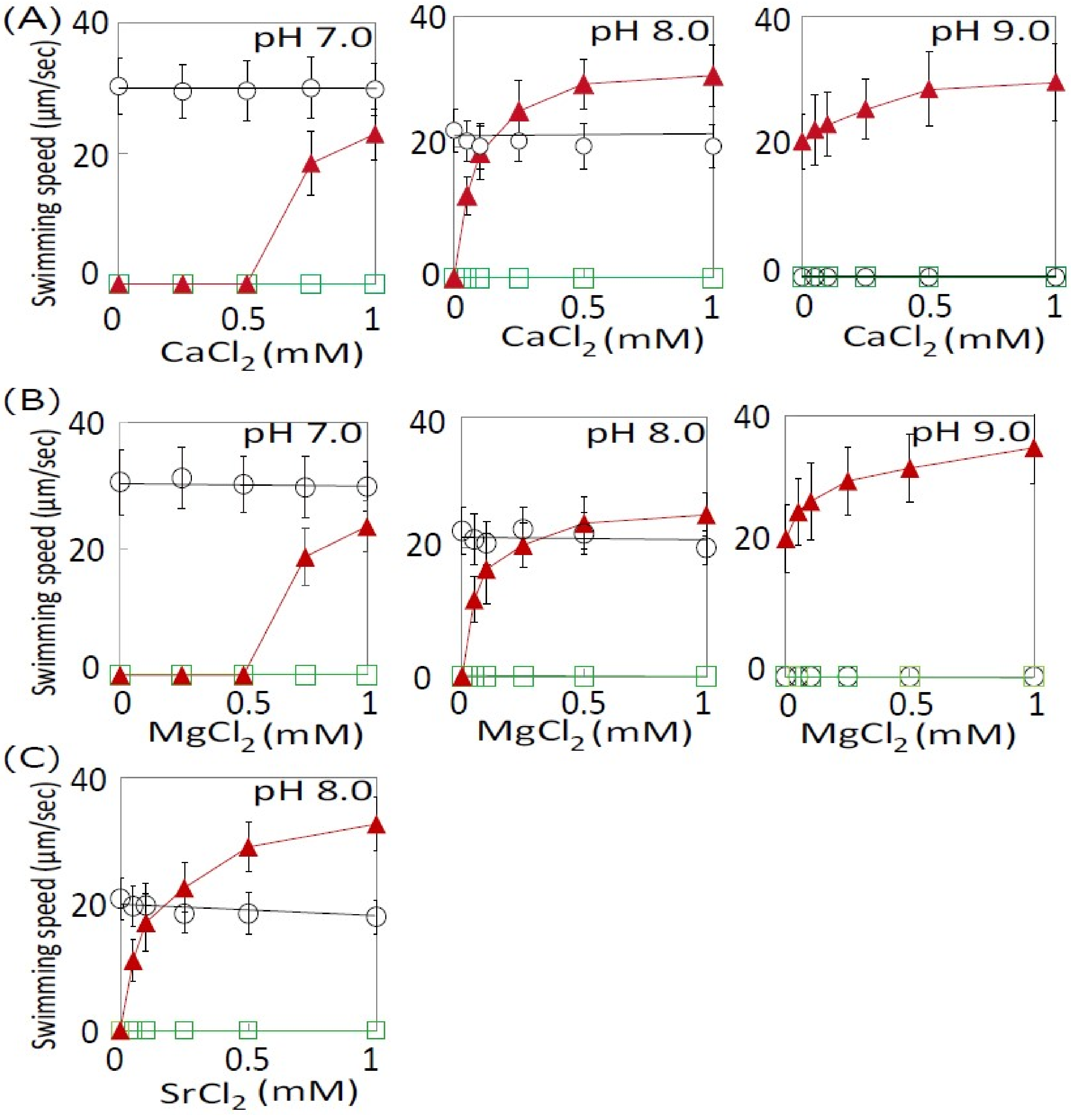


**Fig. S1. Effect of lower concentration of divalent cations on swimming speed of *Paenibacillus* sp. TCA20, *E. coli*, and *B. pseudofirmus* OF4.** Swimming speeds of *Paenibacillus* sp. TCA20, *E. coli*, and *B. pseudofirmus* OF4 cells were measured in 30 mM Tris-HCl containing less than 1 mM $CaCl_2$ (A), $MgCl_2$ (B), or $SrCl_2$ (C) concentrations. The results represent the average swimming speed of 30 independent cells of three independent experiments. The error bars indicate standard deviations.

**Table S1.** Bacterial strains and plasmids used in this study.

| Strains or plasmid | Description | Source or reference |
|---|---|---|
| Strain | | |
| *Escherichia coli* | | |
| DH5αMCR | $F^-$ *mcrA*Δ1 (*mrr-hsd RMS-mcrBC*) Φ80*dlacZ* Δ(*lacZYAargF*) *U169 deoR recA1 endA1 supE44* λ*thi-1 gyr-496 relA*1 | Stratagene |
| *Paenibacillus* sp. | | |
| TCA20 | Wild type | This study |
| *Bacillus pseudofirmus* | | |
| OF4 | Wild type | (1) |
| *Bacillus subtilis* | | |
| BR151MA | *lys3 trpC2* (wild type) | (2) |
| ΔABΔPS | *lys3 trpC2* Δ*motAB* Δ*motPS* | (3) |
| BS-AB | ΔABΔPS *lacA*::$P_{xylA}$-*motAB* from BR151MA | (4) |
| BS-PS | ΔABΔPS *lacA*::$P_{xylA}$-*motPS* from BR151MA | (4) |
| OF4PS | ΔABΔPS *lacA*::$P_{xylA}$-*motPS* from OF4 | This study |
| TCA-AB1 | ΔABΔPS *lacA*::$P_{xylA}$-*motAB1* from TCA20 | This study |
| TCA-AB2 | ΔABΔPS *lacA*::$P_{xylA}$-*motAB2* from TCA20 | This study |
| ΔABPSΔKQ | ΔABΔPS Δ*ykoK* Δ*yfjQ* | This study |
| ΔΔTCA-AB1 | ΔABPSΔKQ *lacA*::$P_{xylA}$-*motAB1* from TCA20 | This study |
| Plasmid | | |
| pGEM-7zf(+) | Cloning vector; $Ap^R$ | Promega |
| pAX01 | *lacA* integration vector with $Em^R$ gene and $P_{xylA}$ promoter upstream of multiple cloning site | (5) |
| pGEM-AB1 | pGEM-7zf(+) + *motAB1* from TCA20 | This study |
| pGEM-AB2 | pGEM-7zf(+) + *motAB2* from TCA20 | This study |
| pAX-$P_{xylA}$-AB1 | pAX01 + $P_{xylA}$-*motAB1* from TCA20 | This study |
| pAX-$P_{xylA}$-AB2 | pAX01 + $P_{xylA}$-*motAB2* from TCA20 | This study |
| pUC18Tc | Cloning vector, $Ap^R$::$Tc^R$ | (3) |
| pUC18Tc-ΔykoK | pUC18Tc+Δ*ykoK* fragment | This study |
| pUC18Tc-ΔyfjQ | pUC18Tc+Δ*yfjQ* fragment | This study |

Table S2. Oligonucleotides used in this study.

| Primer | Sequence (5′→3′) [a] | Accession number and corresponding sequence [b] |
|---|---|---|
| PUmotAB1-SacII-F | gttcccgCGGattatactcggttcatg | BBIW01000007.1<br>(13736-13762) |
| PUmotAB1-SacII-R | ccatcCcgcGGtaaaaatcaggatgg | BBIW01000007.1<br>(15458-15483) |
| PUmotAB2-SacII-F | aacCCgCggatatcttgaaaggattcag | BBIW01000023.1<br>(33114-33130) |
| PUmotAB2-SacII-R | caaagccGcGGacaggattggaggc | BBIW01000023.1<br>(34800-34824) |
| BS-YkoK-CM-1 | GAAATTTCCGCAAAAGATGGACGC | CP010052.1<br>(1395250-1395273) |
| BS-YkoK-CM-2 | GGCTCGCAGTTGAGACGGACGTACCTCCTCTACGGAGACG | CP010052.1<br>(1395998-1396017)<br>(1397391-1397410) |
| BS-YkoK-CM-3 | CGTCTCCGTAGAGGAGGTACGTCCGTCTCAACTGCGAGCC | CP010052.1<br>(1395998-1396017)<br>(1397391-1397410) |
| BS-YkoK-CM-4 | CGGTATTGTCCGTTTTGAACCG | CP010052.1<br>(1398073-1398094) |
| BS-YfjQ-CM-1 | CGAACATGAGGACGTTTTGCACGG | CP010052.1<br>(873101-873124) |
| BS-YfjQ -CM-2 | GGCTTACAACAAAAAAGAACCCTCCACCTGCCATTATATC | CP010052.1<br>(872323-872342)<br>(871322-871340) |
| BS-YfjQ -CM-3 | GATATAATGGCAGGTGGAGGGTTCTTTTTTGTTGTAAGCC | CP010052.1<br>(872323-872342)<br>(871322-871340) |
| BS-YfjQ -CM-4 | GCCCTAAAGACATTTTGAAGCCG | CP010052.1<br>(870568-870546) |

[a] Nucleotides that were added to introduce point mutations are shown by a capital letter.[b] Minus strand is underlined.

## References


1. Guffanti AA, *et al.* (1986) Isolation and characterization of new facultatively alkalophilic strains of *Bacillus* species. *J. Bacteriol.* 167(3):766-773.
2. Grundy FJ & Henkin TM (1991) The *rpsD* gene, encoding ribosomal protein S4, is autogenously regulated in *Bacillus subtilis*. *J Bacteriol* 173(15):4595-4602.
3. Takahashi Y, Koyama K, & Ito M (2014) Suppressor mutants from MotB-D24E and MotS-D30E in the flagellar stator complex of *Bacillus subtilis*. *J. Gen. Appl. Microbiol.* 60(4):131-139.
4. Takahashi Y & Ito M (2014) Mutational analysis of charged residues in the cytoplasmic 542 loops of MotA and MotP in the *Bacillus subtilis* flagellar motor. *J. Biochem.* 156(4):211-220.
5. Ireton K, Rudner DZ, Siranosian KJ, & Grossman AD (1993) Integration of multiple developmental signals in *Bacillus subtilis* through the Spo0A transcription factor. *Genes Dev.* 7(2):283-294.
